# The tradeoffs between persistence and mutation rates at sub-inhibitory antibiotic concentrations in *Staphylococcus aureus*

**DOI:** 10.1128/spectrum.02479-24

**Published:** 2025-03-04

**Authors:** Alysha S. Ismail, Brandon A. Berryhill, Teresa Gil-Gil, Joshua A. Manuel, Andrew P. Smith, Fernando Baquero, Bruce R. Levin

**Affiliations:** 1 Department of Biology, Emory University, Atlanta, Georgia, USA; 2 Program in Microbiology and Molecular Genetics, Graduate Division of Biological and Biomedical Sciences, Laney Graduate School, Emory University, Atlanta, Georgia, USA; 3 Servicio de Microbiología, Hospital Universitario Ramón y Cajal, Instituto Ramón y Cajal de Investigación Sanitaria, and Centro de Investigación Médica en Red de Epidemiología y Salud Pública (CIBERESP), Madrid, Spain; 4 Emory Antibiotic Resistance Center, Emory University, Atlanta, Georgia, USA; Icahn School of Medicine at Mount Sinai, New York, New York, USA

**Keywords:** antibiotics, minimum inhibitory concentration, pharmacodynamics, population biology, antibiotic resistance mutation rate, bacterial persistence, antibiotic heteroresistance, *Staphylococcus aureus*

## Abstract

The rational design of the antibiotic treatment of bacterial infections employs these drugs to reach concentrations that exceed the minimum needed to prevent the replication of the target bacteria. However, within a treated patient, spatial and physiological heterogeneity promotes antibiotic gradients such that the concentration of antibiotics at specific sites is below the minimum needed to inhibit bacterial growth. Here, we investigate the effects of sub-inhibitory antibiotic concentrations on three parameters central to bacterial infection and the success of antibiotic treatment, using *in vitro* experiments with *Staphylococcus aureus* and mathematical and computer-simulation models. Our results, using drugs of six different classes, demonstrate that exposure to sub-inhibitory antibiotic concentrations alters bacterial growth dynamics, increases the mutation rate to antibiotic resistance, and decreases the production of persister cells thereby reducing persistence levels. Understanding this trade-off between mutation rates and persistence levels resulting from sub-inhibitory antibiotic exposure is crucial for optimizing, and mitigating the failure of, antibiotic therapy.

**IMPORTANCE:**

Much of the research on antibiotics and antibiotic treatment has focused on drug concentrations sufficient to prevent the growth of bacteria. These concentrations, however, are not always reached everywhere in the body. Here, we look at the effects of exposure to these low concentrations of antibiotics on the common clinically important pathogen *Staphylococcus aureus*. We confirm a previous finding that sub-inhibitory antibiotic exposure decreases the total growth and the growth rate of the bacteria. Moreover, we demonstrate that the level of persistence, an important mechanism for bacteria to survive antibiotics, is decreased due to sub-inhibitory exposure. However, we find that the rate of generation of resistant mutants is substantially increased. Taken together, these results reveal an important trade-off that emerges as a consequence of bacteria being exposed to sub-inhibitory concentrations of antibiotics.

## INTRODUCTION

In the rational design of antibiotic therapy, drugs are administered such that the concentration of the treating drug exceeds the threshold needed to prevent the replication of the target pathogen (1). However, in a treated individual, the concentration of an antibiotic within the body varies across different anatomical regions due to factors such as variations in vascularization and the pharmacokinetics (PK) of the treating antibiotic (2). Notably, even though antibiotics are administered such that the concentration of the drug exceeds the Minimum Inhibitory Concentration (MIC), they are often present at sub-inhibitory concentrations over time throughout the body (3). Despite this, almost all studies on the pharmacodynamics of antibiotics focus on super-inhibitory concentrations, ignoring the effects of sub-inhibitory concentrations of antibiotics on bacterial populations.

In this study, we utilize a laboratory strain of the clinically relevant pathogen *Staphylococcus aureus* (4) to examine the impact of exposure to sub-inhibitory concentrations of six antibiotic classes on growth dynamics, mutation rates, and the level of persistence. Persistence is a phenotypic change in which a fraction of quiescent bacterial cells survive treatment with a super-inhibitory concentration of an antibiotic (5). Importantly, this study seeks to determine how pre-exposure to sub-inhibitory concentrations of antibiotics from multiple classes affects mutation rates and persistence when the cultures are subsequently exposed to super-inhibitory concentrations of different drugs. By testing a wide range of antibiotics, we aim to demonstrate that the impact of pre-exposure to sub-inhibitory concentrations on mutation rates and persistence is not specific to a single antibiotic but is a generalizable effect. In a previous study with *Escherichia coli*, we demonstrated that exposure to sub-inhibitory concentrations of antibiotics results in a decrease in the growth rate along with the maximum bacterial density achieved, as well as an increase in the lag phase (the time before the bacterial population begins to replicate) (6). We confirm the generality of those findings here. Moreover, other studies have established that super-inhibitory antibiotic concentrations can elevate the mutation rate for resistance to other drugs (7). We have found that this phenomenon extends to sub-inhibitory antibiotic concentrations as well. Finally, we provide evidence that pre-exposure to sub-inhibitory concentrations of antibiotics decreases the level of persistence.

## RESULTS

### The generality of sub-inhibitory concentration of antibiotics on bacterial growth dynamics

**Fig 1:**
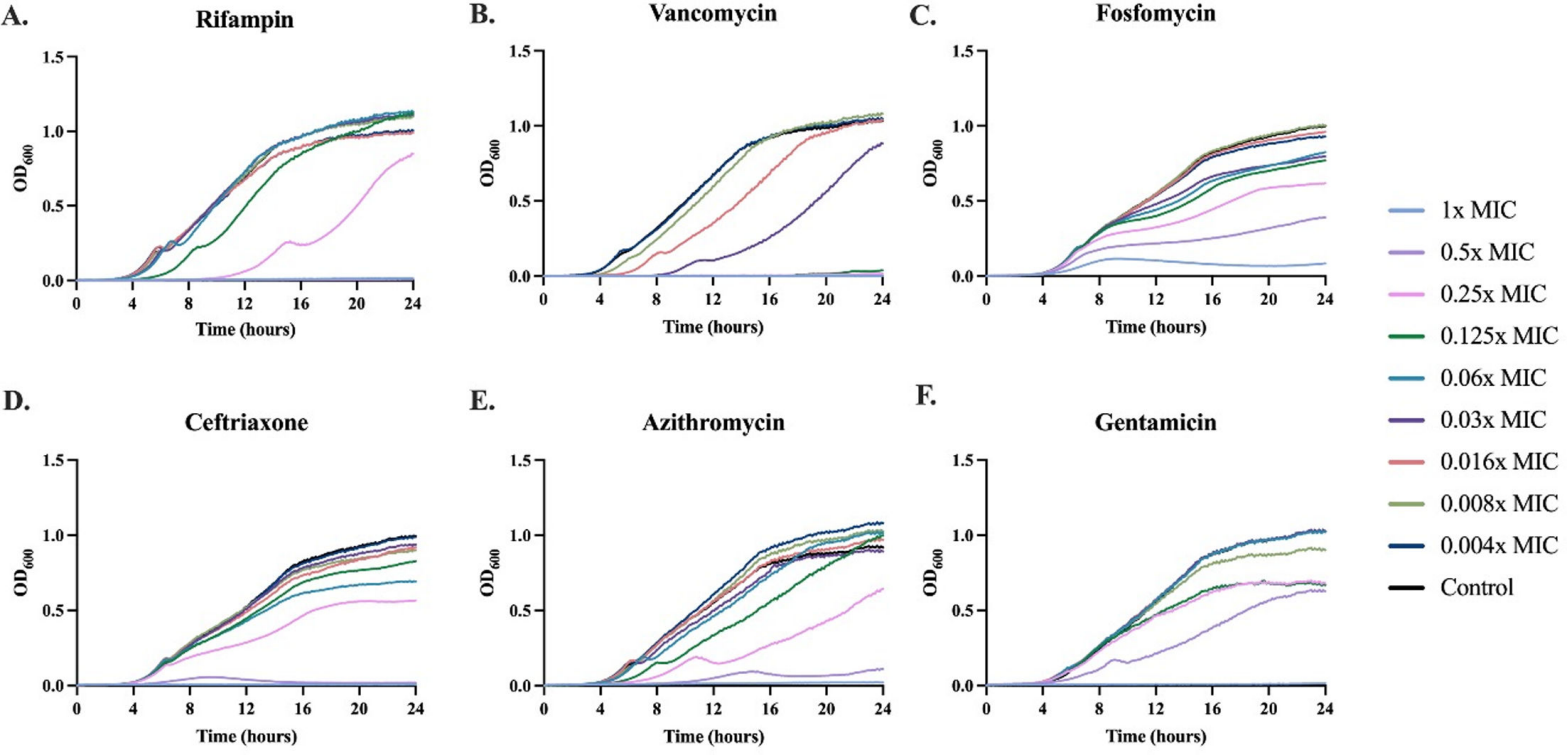
Growth dynamics of *S. aureus* with varying antibiotics and concentrations. Changes in the optical density at 600 nm (OD600) exposed to different concentrations of six classes of drugs (A–F). Lines represent the average of five technical replicates. Each concentration is given as a fraction of the MIC shown in Table S1: 1× (light blue), 0.5× (light purple), 0.25× (pink), 0.125× (green), 0.06× (blue), 0.03× (purple), 0.016× (red), 0.008× (light green), 0.004× (dark blue), with a drug-free control shown in black.

To determine the effects of exposure to sub-inhibitory concentrations of antibiotics on the growth dynamics of bacteria, we follow the changes in the optical densities (ODs) of *Staphylococcus aureus* Newman exposed to sub-inhibitory concentrations of antibiotics from six different classes (Fig. 1). The growth dynamics of *S. aureus* Newman vary among the drugs for all six antibiotics; however, there is a clear concentration-dependent variation in the maximum growth rate (Fig. S1), maximum optical density (Fig. S2), and lag time (Fig. S3). These results are consistent with those previously observed for *Escherichia coli* (6), demonstrating that the results obtained previously are not restricted to Gram-negative bacteria (8).

### The effects of exposure to sub-inhibitory concentration of antibiotics on the mutation rate

#### Null model of mutation rate

To explore the intrinsic variation in the estimation of mutation rates, we use a mathematical and computer-simulation model that employs the Monte Carlo process to generate mutants (Supplemental Text and Supplemental Equations 1–4) (9). Shown in Table 1 are five independent runs of this model each with 20 independent replicates. Though there is variation in the estimated mutation rate between runs, this variation is not statistically significant.

**TABLE 1:** Variation in mutation rates estimated from a Monte Carlo simulation of random mutation

	Null model mutation rate predictions
Trial 1	2.96 × 10^−9^
Trial 2	3.44 × 10^–9^
Trial 3	3.43 × 10^–9^
Trial 4	2.20 × 10^–9^
Trial 5	2.98 × 10^–9^

#### Changes in the mutation rate due to sub-inhibitory drug pre-exposure

To determine the effect sub-inhibitory pre-exposure has on the mutation rate to antibiotic resistance, we exposed *S. aureus* Newman to the concentration of the six drugs previously used that did not change the maximum stationary phase density. After 24 h of pre-exposure, we performed a Luria–Delbruck fluctuation test to determine the mutation rate to streptomycin resistance (Table 2) (10). This method involves inoculating multiple parallel cultures to quantify the occurrence of spontaneous mutations, distinguishing mutation events from adaptive responses. Notably, pre-exposure to sub-inhibitory concentrations of antibiotics significantly increased the mutation rate to streptomycin resistance, a result unanticipated by the null model.

**TABLE 2:** Mutation rates to streptomycin resistance in *S. aureus* pre-exposed to different antibiotics ^*a*^

	*S. aureus* Newman	JE2 Δ*recA*
Control	5.05 × 10^−9^ ± 8.98 × 10^−10^	3.53 × 10^−8^ ± 4.08 × 10^−9^
Rifampin	4.96 × 10^−8^ ± 1.25 × 10^−8*^	3.45 × 10^−8^ ± 6.35 × 10^−9^
Vancomycin	4.29 × 10^−8^ ± 1.44 × 10^−8**^	3.17 × 10^−8^ ± 6.88 × 10^−9^
Fosfomycin	4.13 × 10^−8^ ± 754 × 10^−9**^	-^*b*^
Ceftriaxone	2.32 × 10^−8^ ± 6.24 × 10^−9**^	-
Azithromycin	6.98 × 10^−8^ ± 9.11 × 10^−9***^	-
Gentamicin	1.80 × 10^−8^ ± 3.51 × 10^−9**^	4.17 × 10^−8^ ± 7.44 × 10^−9^

^*a*^ *P< 0.05, **P < 0.005, ***P < 0.0005.

^*b*^ -, no data.

To elucidate the contribution of the generalized bacterial stress response, known as the SOS response, to the increase in mutation rate, we repeated the above experiments with a strain lacking the *recA* gene, the major constituent of the SOS response (11). When this knockout strain was pre-exposed to the same fraction of the MIC of each drug, there was no evidence of a significant increase in the mutation rate (Table 2). The *recA* knockout strain, obtained from the *S. aureus* JE2 strain, was resistant to fosfomycin, ceftriaxone, and azithromycin; thus, these antibiotics could not be used for the pre-exposure of this strain (Table S1). In principle, RecA deletion should reduce the mutation rate, and thus, polymerase V activation and SOS mutagenesis do not take place (12). However, this fact essentially applies to DNA-damaging agents like fluoroquinolones (13) and not so much to the antibiotics that target the cell wall or ribosomal function. The JE2 strain was found to have a higher baseline mutation rate than Newman (4.01 × 10^−8^ ± 8.87 × 10^−9^). However, when pre-exposed to sub-inhibitory concentrations of antibiotics, JE2 still exhibited a 10-fold increase in the mutation rate to streptomycin (*P* = 0.008, *n* = 20).

Streptomycin was the only drug used to estimate the mutation rate, although other antibiotics were tested. For this experiment, the mechanism of resistance for the chosen antibiotics is a single-point mutation, which significantly limits the classes of drugs that could be used. The fluoroquinolones were found to have too low of a baseline mutation rate, such that it was below the limit of detection. Interestingly, *S. aureus* Newman was found to be heteroresistant to the quinolone nalidixic acid, while it was not heteroresistant to the fluoroquinolone ciprofloxacin (Fig. S4).

### The effects of exposure to sub-inhibitory concentrations of antibiotics on the level of persistence

#### Null model of persistence

**Fig 2:**
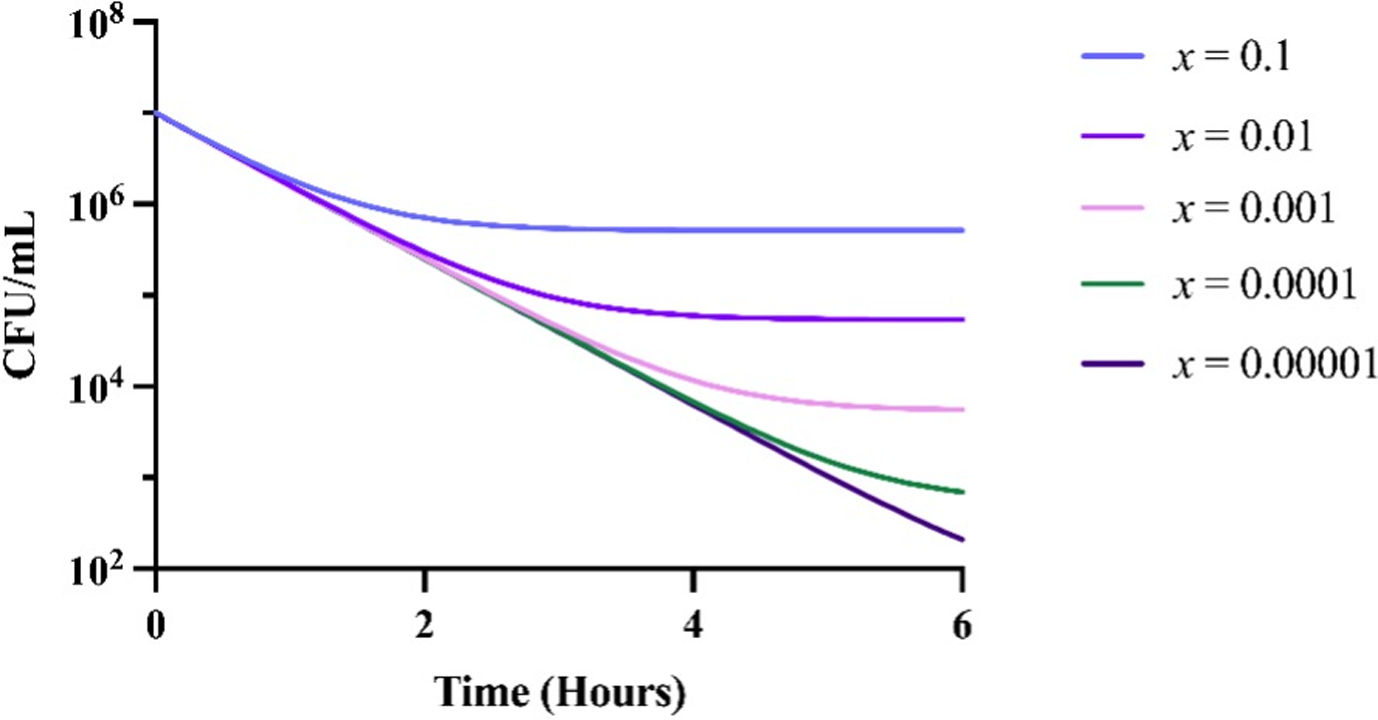
Predicted changes in the total cell density of a bacterial population capable of producing persister cells to a bactericidal antibiotic. These simulations assume all parameters are equal between runs except for the parameter *x*, the rate constant of persister cell generation. The other parameters used for this simulation are *A* = 5.0, *v*_*S*_ = 2.0, *v*_*P*_ = 0, *v*_MIN_ = −3.0, *e* = 5 × 10^−7^, MIC = 1.0, and *r* = 1,000.

To determine the effect that the rate of persistence generation has on the final level of persistence, we employed a mathematical and computer-simulation model of persistence with differing rates of persister cell generation (Supplemental Text and Supplemental Equations 5–8). In Fig. 2, we show that a higher rate of persister cell generation results in a higher level of persistence at 6 h, such that in a time-kill experiment the total number of surviving cells would be higher in a rate-dependent manner.

#### Changes in the level of persistence due to sub-inhibitory drug pre-exposure

To determine the effect that sub-inhibitory pre-exposure has on the level of persistence, we first had to select drugs for which *S. aureus* Newman shows persistence—which is shown on time–kill curves as cells that survive super-inhibitory drug exposure but do not replicate and do not have an increased MIC. In Fig. S5, we show that daptomycin and tobramycin, two highly bactericidal antibiotics, both have differing levels of persistence, whereas ciprofloxacin, tetracycline, and streptomycin do not exhibit clear evidence for persistence at the tested concentrations (14). We chose 6× MIC for tobramycin and 4× MIC for daptomycin to perform subsequent time–kill curves to maximize the differences in the levels of persistence. To ensure the drug-exposed survivors were due to persistence and not some other phenomenon, such as tolerance or resistance, single colonies from the last time point of the time–kills were selected, and the time–kill was repeated. The time–kill curves generated from these selected persister colonies were nearly identical, both qualitatively and quantitatively, to those in Fig. S5. This consistency confirms that the surviving cells were true persisters (Fig. S6). MICs were performed on the cells surviving the time–kills, and their MIC was found to be the same as the parental strain, providing evidence for persistence rather than resistance.

**Fig 3:**
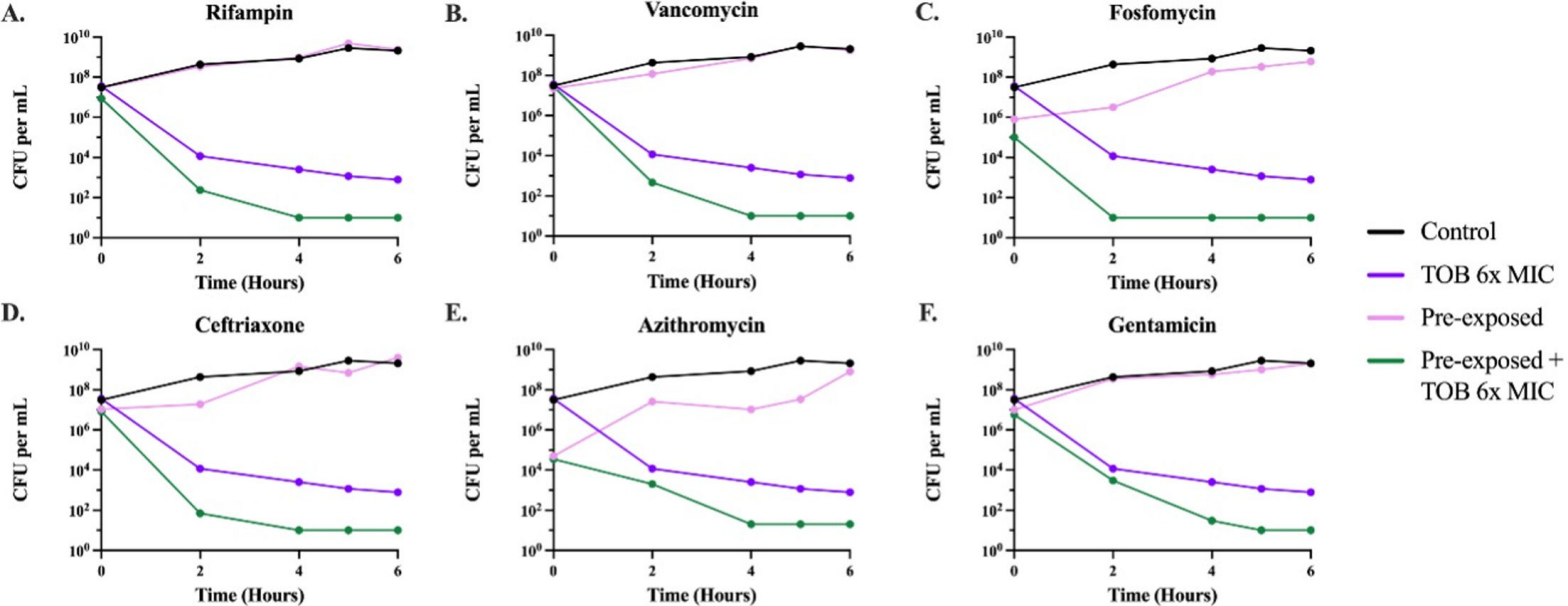
Time–kill experiments with tobramycin. Six-hour time–kill curves were performed with 6× the MIC of tobramycin (Table S1). Cultures were either pre-exposed for 24 h or not pre-exposed to sub-inhibitory concentrations of one of the six antibiotics (A–F); from there, either the cultures were allowed to grow in the absence or presence of tobramycin. Lines represent the following: no pre-exposure, no tobramycin (black); no pre-exposure, with tobramycin (purple); pre-exposure, no tobramycin (pink); and pre-exposure, with tobramycin (green). The persistence level at the 6 h time point is statistically different (*P* < 0.00001) between the pre-exposure with tobramycin (green) and the no pre-exposure with tobramycin (purple).

**Fig 4:**
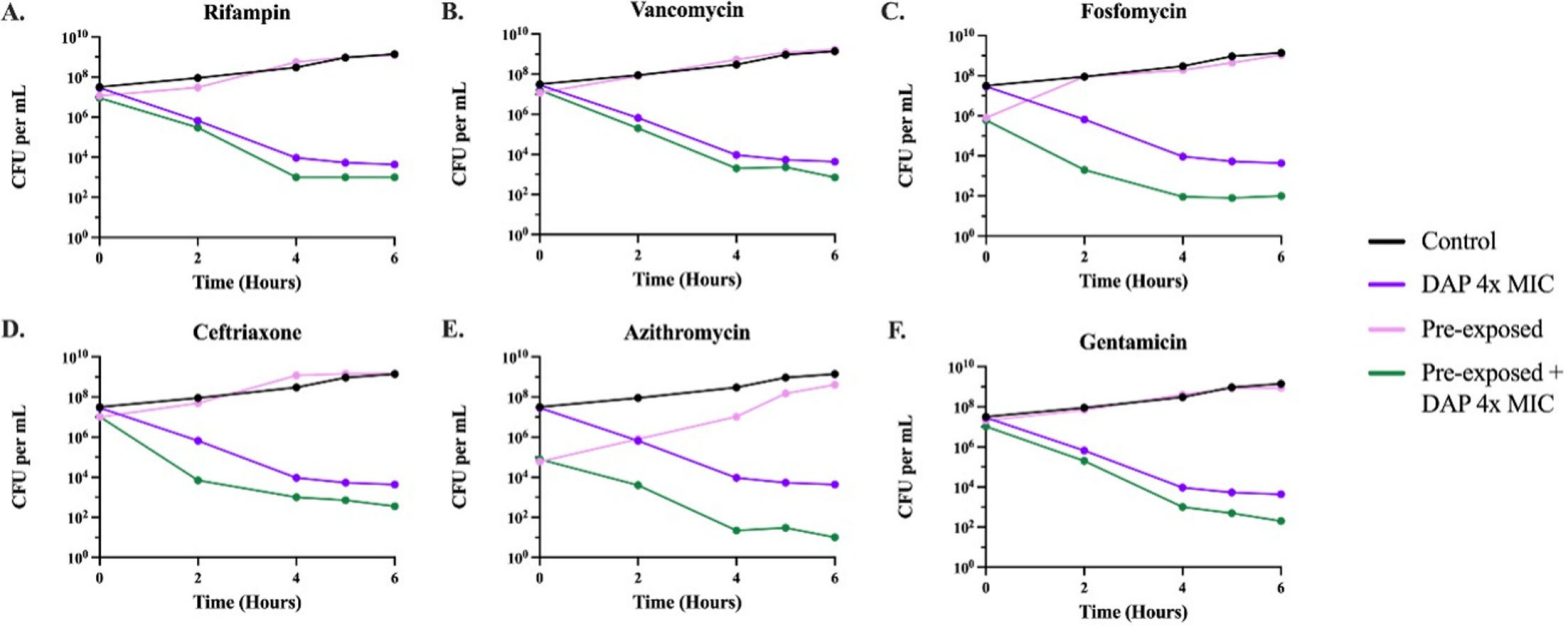
Time–kill experiments with daptomycin. Six-hour time–kill curves were performed with 4× the MIC of daptomycin (Table S1). Cultures were either pre-exposed for 24 h or not pre-exposed to sub-inhibitory concentrations of one of the six antibiotics (A–F); from there, the cultures were allowed to grow in either the absence or presence of daptomycin. Lines represent the following: no pre-exposure, no daptomycin (black); no pre-exposure, with daptomycin (purple); pre-exposure, no daptomycin (pink); and pre-exposure, with daptomycin (green). The persistence level at the 6-h time point is statistically different (*P* < 0.00001) between the pre-exposure with daptomycin (green) and the no pre-exposure with daptomycin (purple).

To elucidate the effects sub-inhibitory pre-exposure has on the level of persistence, we performed time–kill experiments with the drugs and concentrations selected above. Cultures were pre-exposed for 24 h to the six antibiotics used in Fig. 1 at sub-inhibitory concentrations, which were shown not to reduce the stationary phase densities. As shown in Fig. 3 and 4, pre-exposure to sub-inhibitory concentrations of the six antibiotics decreased the levels of persistence to both tobramycin and daptomycin. Variation in the initial density occurred due to the reduced densities generated by exposure to sub-inhibitory concentrations of the drugs.

#### Changes in metabolic activity due to sub-inhibitory drug pre-exposure

**Fig 5:**
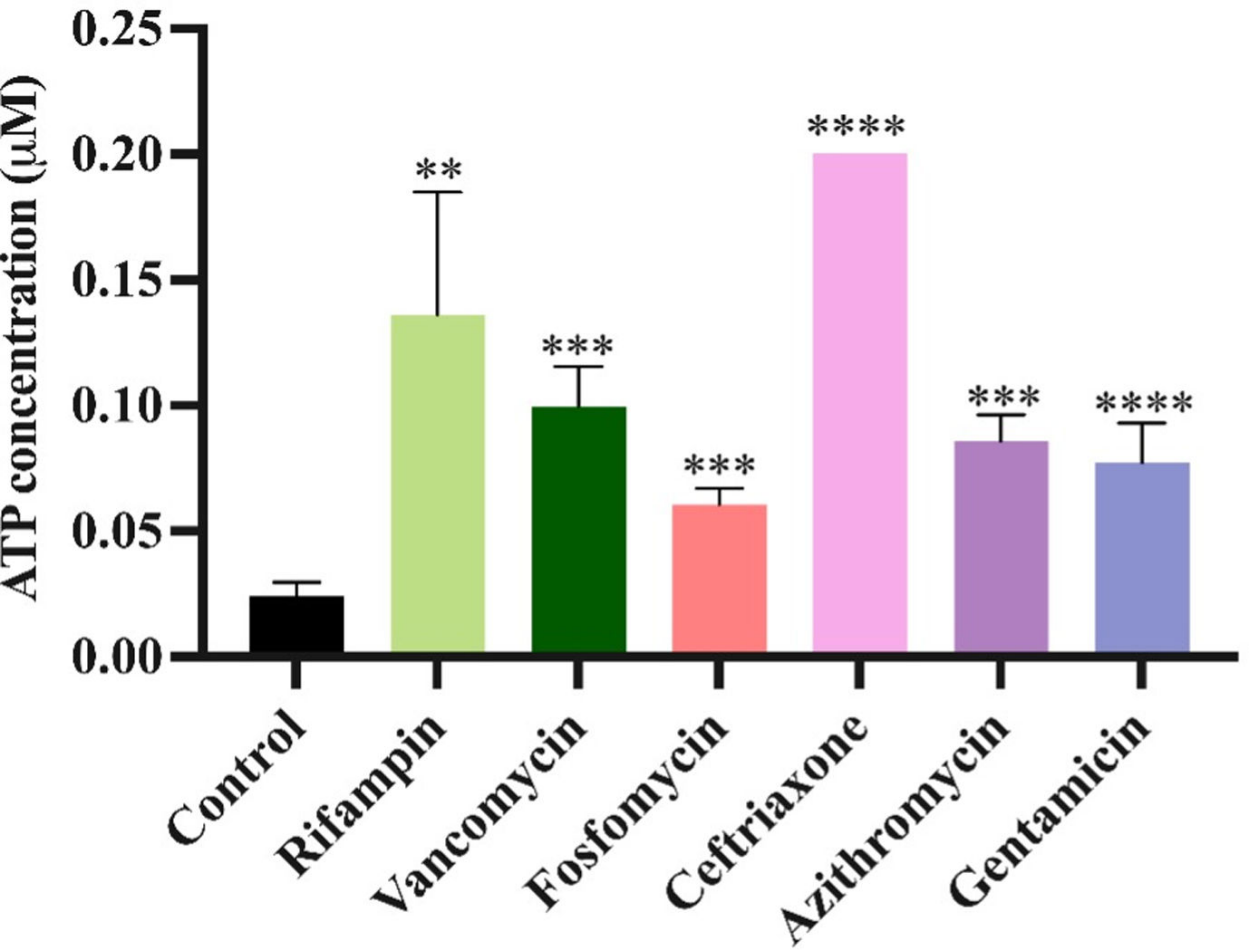
ATP determination. Cultures were either pre-exposed or not pre-exposed to sub-inhibitory concentrations of one of the six antibiotics: From there, the amount of ATP in these cultures was experimentally estimated after 24 h of pre-exposure via luminescence at 560 nm. The error represents the standard deviation of measurements obtained from three independent biological replicates. **P < 0.005, ***P < 0.0005, ****P < 0.00005.

Persister cells enter a state of dormancy in which they reduce their metabolic activity. Accordingly, if metabolism is increased, persistence levels will decrease (15). To evaluate the effect that the pre-exposure to sub-inhibitory concentrations of antibiotics has on bacterial metabolic activity, we measured the intracellular amount of ATP via a luminescence assay. In Fig. 5, we show that pre-exposure to sub-inhibitory concentrations of the selected antibiotics increased the ATP levels, indicating a higher metabolic rate that may account for the results in Fig. 3 and 4.

## DISCUSSION

Antibiotics are prescribed to patients at concentrations designed to exceed the minimum concentration necessary to prevent the replication of the target pathogen (16). Therefore, the MIC is the dominant and often the unique pharmacodynamic parameter used to design antibiotic treatments (17). However, *in vivo* conditions introduce significant variability in factors such as local bacterial concentration at the infection site, replication rate, nutrient availability, and the immune response (18). Moreover, though the antibiotic is administrated at super-inhibitory concentrations, this concentration may not be reached in all, or even most, locations of the body, including the infection sites (19). This means treatment occurs at gradients of antibiotic concentrations throughout the body, including antibiotic concentrations insufficient to kill or prevent the replication of the infecting bacteria (20).

Previous studies have revealed that exposing *E. coli* to sub-inhibitory concentrations of antibiotics leads to decreasing both maximum growth rate and maximum optical density while increasing the lag phase of growth (6). Our results here confirm that this phenomenon applies to *S. aureus* as well. These changes are consistent through all six classes of drugs tested where a concentration-dependent response is observed; as the concentration of the antibiotic increases, so does the degree of impairment of the growth dynamics. These results show that significant antibacterial activity occurs at sub-inhibitory concentrations, in some cases, exceptionally lower than the MIC, suggesting that antibiotics may have clinical utility at sub-inhibitory concentrations. This may explain why infections can be successfully treated despite being located in sites where super-inhibitory antibiotic concentrations are not achieved. Apart from locational heterogeneity, sub-inhibitory antibiotic concentrations can also occur due to suboptimal dosing, extending the time between doses, and using partially inactivated drugs due to inappropriate storage.

Along with the changes in growth dynamics, sub-inhibitory exposure may lead to physiological changes in the bacteria (21). When bacteria are exposed to super-inhibitory concentrations of antibiotics, resistant mutants in the population will be able to survive and replicate in the presence of this selective pressure due to mutations (22, 23). Mutation rates, including those of antibiotic resistance, are not fixed. One pathway that modulates mutation rates is the SOS response, which is nearly ubiquitous in bacteria (24). This response plays a vital role in DNA repair and enables survival under physiological stress. Several external factors can lead to the activation of the SOS response (25). Our results illustrate that one of these factors is exposure to sub-inhibitory concentrations of antibiotics (Table 2). The major regulator of the SOS response is RecA (11). In *S. aureus*, there are two major pathways involved in this response: the LexA-dependent pathway, which results in the expression of the UmuC error-prone polymerase, and the RexAB-dependent pathway, which results in the formation of small-colony variants (26–28). Taken together, activation of both pathways results in an increase in the mutation rate of at least one order of magnitude, as we have shown in Table 2. As expected, when *recA* is knocked out, these pathways cannot be activated, and pre-exposure to sub-inhibitory concentrations of antibiotics does not lead to a change in the mutation rate. The actual mutation rate may be higher than estimated here due to the fact that, in the pre-exposed cultures, some death may have occurred. However, as shown in Fig. S7, there is a high correlation between the measures of colony-forming units (CFUs; live cells) and OD (total cells which include dead cells), and therefore, the mutation rate may be slightly higher. Stated another way, due to pre-exposure to sub-inhibitory concentrations of antibiotics, the mutation rates presented here may be slightly underestimated, but still, the trend of increasing mutation rates under drug pre-exposure still holds. We also observed that *S. aureus* Newman is heteroresistant to the quinolone nalidixic acid, while it is not heteroresistant to the fluoroquinolone ciprofloxacin.

Another phenomenon that could arise from pre-exposure to sub-inhibitory concentrations of antibiotics is changes in persistence levels. Persistence is a temporary phenotypic change in which the majority of the population is susceptible to antibiotics and a minority population is capable of surviving exposure to antibiotics without an increase in the MIC (29, 30). Persister cells can survive antibiotic treatment by entering a dormant or slow-growing state, due to several possible mechanisms (31, 32). Different environmental factors can change the frequency of generation of persister cells in a bacterial population; our results here show that one of these factors is the exposure to antibiotics—in this case, sub-inhibitory levels of six distinct antibiotics. When bacteria are confronted with sub-inhibitory levels of antibiotics before encountering super-inhibitory concentrations of other drugs, it triggers metabolic changes, which decrease the rate of generation of these persister cells. Our results further demonstrate that these metabolic changes occur due to exposure to sub-inhibitory concentrations of antibiotics, which is shown by a higher intracellular ATP concentration (Fig. 5). This increase in metabolic activity opposes the dormancy that defines persistence, therefore, leading to a lower rate of persister cell formation when the bacterial populations are then exposed to super-inhibitory concentrations of other drugs.

Consistent with this hypothesis, exposure to sub-inhibitory concentrations of antibiotics for a short term (the duration of the lag period) also decreased the level of persistence (Fig. S8). Unexplored, but testable, implications also arise from this increase in metabolic activity. The observation can be interpreted on the basis of the Julian Davies’ concept of “hormesis,” meaning that differences in the concentration (quantity) of a drug might result in qualitative differences in the response of bacterial cells to antibiotics (33). Inhibitory (particularly bactericidal) antibiotic concentrations promote a rapid induction in oxygen consumption indicative of elevated respiration, but the production of reactive oxygen species (ROS) reduces tricarboxylic acid cycle enzymes, lowering the energy for metabolism and ATP formation, with an increased level of antibiotic persistence (34, 35). On the contrary, sub-inhibitory concentrations increase respiration (via cytochrome bd-1), reducing ROS formation, boosting metabolism, enhancing ATP formation, and consequently decreasing the level of antibiotic persistence (36). Conceivably, toxins and other virulence factors are also upregulated by exposure to sub-inhibitory concentrations of antibiotics.

These results contribute to our understanding of the interaction between bacterial mutation, persistence, and antibiotics as an academic matter; however, there are serious clinical implications that follow these findings as well (37). The administration of a first line of antibiotic therapy will create a gradient of antibiotic concentrations within the body. If this first treatment fails, and a secondary line of treatment is administered, the increase in mutation rate produced in response to the sub-inhibitory concentrations in different body locations could lead to the generation of resistant mutants, which could then result in treatment failure that would not otherwise have occurred. On the other hand, we show that persistence would be reduced wherever there was pre-exposure to antibiotics. This ability to persist is an important attribute for bacterial populations when conditions are unfavorable for their survival. As a result, pre-exposure to sub-inhibitory concentrations of antibiotics reducing persistence levels could enhance second-line treatment efficacy, improving the effectiveness of super-inhibitory concentrations of the antibiotic used in therapy and, therefore, reducing the risk of recurrent infections (38). These results are especially salient in chronic and recurrent infections such as those involving biofilms (39). Ultimately, these findings boil down to one important trade-off that has real-world impacts in the clinic, that is, a trade-off between higher mutation rates and lower persistence levels resulting from previous exposure to sub-inhibitory concentrations of antibiotics.

## MATERIALS AND METHODS

### Growth media

All experiments were conducted in Muller Hinton II (MH II) Broth (90922-500G) obtained from Millipore. All bacterial quantification was done on Lysogeny Broth (LB) agar (244510) plates obtained from BD. E-tests were performed on MH agar plates made from MH broth (M391-500g) with 1.6% agar obtained from HiMedia.

### Growth conditions

Unless otherwise stated, all experiments were conducted at 37°C with shaking.

### Bacterial strains

All experiments were performed with *Staphylococcus aureus* Newman obtained from Bill Schafer of Emory University. JE2 Δ*recA* and JE2 from the Nebraska Transposon Mutant Library (40) were obtained from Joanna Goldberg of Emory University and are derivatives from the MRSA USA300 strain.

### Antibiotics

Streptomycin (S6501), sulfamethoxazole (S6377), vancomycin (V1130), ceftriaxone (C5793), fosfomycin (P5396), and daptomycin (D2446) were all obtained from Sigma-Aldrich. Tobramycin (T1598) was obtained from Spectrum. Azithromycin (3771) was obtained from TOCRIS. Ciprofloxacin (A4556) was obtained from AppliChem Panreac. Gentamicin (BP918-1) and rifampin (BP2679-1) were obtained from Fisher. Nalidixic acid (KCN23100) was obtained from PR1MA. Tetracycline (T17000) was obtained from Research Products International. All E-test strips were obtained from Biomérieux.

### Sampling bacterial densities

The densities of bacteria were estimated by serial dilution in 0.85% saline, and the total density of bacteria was estimated on LB plates with 1.6% agar.

### Growth rate estimation

Exponential growth rates were estimated from changes in OD600 in a Bioscreen C. For this, 24-h overnight cultures were diluted in MHII to an initial density of approximately 10^5^ cells/mL. Five technical replicates were performed for each condition in a 100-well plate. The plates were incubated at 37°C and shaken continuously. Estimates of the OD600 were made every 5 min for 24  h. Normalization was performed, and then, means and standard deviations of the maximum growth rate, lag time, and maximum OD were calculated using an R Bioscreen C analysis tool, which is accessible online at https://josheclf.shinyapps.io/bioscreen_app.

### The correlation between CFU and OD

Ten separate overnights of *S. aureus* Newman with 10^5^ CFU/mL of cells were exposed to either sub-inhibitory or inhibitory concentrations of antibiotics (rifampin, vancomycin, fosfomycin, ceftriaxone, gentamicin, azithromycin) at concentration fractions of 1, 0.5, 0.25, 0.125, 0.06, 0.03, 0.016, 0.008, 0.004, and 0× MIC. After a 24-h incubation, the 10 flasks were then plated for CFU, and the optical density was estimated using a spectrophotometer at 600 nm.

### Minimum inhibitory concentration estimation via broth microdilution

MICs were determined according to the Clinical and Laboratory Standards Institute guidelines, deviating only in the choice of media (41). Briefly, 96-well plates with twofold dilutions of antibiotics in MHII media were prepared and inoculated with 10^5^ bacteria/mL. An extended gradient was created by combining three sets of twofold serial dilutions from three starting antibiotic concentrations. The plates were incubated at 37°C with shaking conditions, and the optical density (OD600) was measured after 24 h.

### Fluctuation tests

Independent overnights of *S. aureus* Newman, JE2 Δ*recA*, and JE2 were either exposed to sub-inhibitory concentrations of antibiotic (0.5× the MIC of rifampin, 0.5× the MIC of vancomycin, 0.25× the MIC of fosfomycin, 0.25× the MIC of ceftriaxone, 0.25× the MIC of gentamicin, or 0.25× the MIC of azithromycin) or grown without antibiotics. These overnights were made in 2 mL of MHII with 10^5^ CFU/mL of cells in the logarithmic phase, then subjected to the pressure of sub-inhibitory concentrations of the chosen drugs and grown overnight. One milliliter of these liquid cultures was then plated on LB plates with 5× the MIC of streptomycin. Initial densities of these cultures were estimated on LB plates. All colonies were then counted 48 h after plating. Experiments were performed with 20 biological replicates, and the mutation rates were calculated as described in References (42, 43) using BZrates.

### Time–kill experiments

Cultures of 10^7^ *S. aureus* Newman were either exposed overnight or for 5 h to sub-inhibitory concentrations of rifampin, vancomycin, fosfomycin, ceftriaxone, azithromycin, or gentamicin at the abovementioned concentrations. Control cultures were grown without antibiotics. After the incubation period, all cultures were diluted in fresh MHII to 10^7^ cells/mL. The cultures were then exposed to super-MIC concentrations of either streptomycin, daptomycin, tetracycline, tobramycin, or ciprofloxacin, and viable cell density was estimated at 0, 2, 4, and 6 h.

### Population analysis profile test

Population analysis profile tests were performed as described in References (44, 45). Briefly, a gradient of nalidixic acid or ciprofloxacin concentrations was added to LB plates. The concentrations were 0, 0.5, 1, 2, 4, 8, 16, and 32 × MIC. Multiple dilutions of *S. aureus* Newman (10^0^–10^−7^) were then plated on every concentration. Colonies were enumerated after 48 h, and the highest dilution with colonies present was recorded. The frequency of surviving cells was calculated by dividing the highest density of cells at each concentration by the number of surviving cells on plates with no antibiotics.

### Numerical solutions (simulations)

For our numerical analysis of the mathematical models detailed in the Supplemental Text, we used Berkeley Madonna, using parameters in the ranges estimated for *S. aureus* Newman. Copies of the Berkeley Madonna program used for these simulations are available at www.eclf.net.

### Statistical analysis

Statistical significance analysis for the time–kill experiments (Fig. 3 and 4) was carried out by ordinary one-way analysis of variance, and the analysis for the ATP assay (Fig. 5) was performed by a paired *t*-test, both using GraphPad Prism (version 10.2.0).

### ATP assay

ATP determination kits were obtained from ThermoFisher Scientific (A22066). To perform the ATP determination, the manufacturer’s provided protocol was followed with the following changes. Overnight cultures either pre-exposed to the antibiotics or not exposed were pelleted, and the pellets were washed with saline. Each experiment was performed in biological duplicate with six technical replicates. Cultures were resuspended in saline and sonicated with a Branson Needle-Tip Sonicator. Post-sonication, cells were centrifuged, and the supernatants were placed in a black 96-well plate and incubated at room temperature for 30 min. After incubation, luminescence was then read at 560 nm.

## SUPPLEMENTAL MATERIAL

Supplemental material spectrum.02479-24-s0001.docx Supplemental text; Fig. S1 to S8; Table S1.

ASM does not own the copyrights to Supplemental Material that may be linked to, or accessed through, an article. The authors have granted ASM a non-exclusive, world-wide license to publish the Supplemental Material files. Please contact the corresponding author directly for reuse.
